# Initial Coverage and Regional Disparities of the National HPV Vaccination Program in Poland: A Cross-Sectional Analysis

**DOI:** 10.3390/healthcare13243281

**Published:** 2025-12-14

**Authors:** Patryk Poniewierza, Marcin Śniadecki, Oliwia Musielak, Afsheen Raza, Yousra Safari, Olga Piątek-Dalewska, Martyna Danielkiewicz, Alicja Mazur, Zofia Amerek, Saqib Raza Khan, Dariusz Grzegorz Wydra

**Affiliations:** 1 Faculty of Medicine, Lazarski University, Swieradowska 43, 02-662 Warsaw, Poland; 2 Department of Obstetrics and Gynecology, Medical University of Gdansk, Smoluchowskiego 17, 80-210 Gdansk, Poland; marcinsniadecki@gumed.edu.pl (M.Ś.);; 3 Memorial MD Zofia Garlicka Clinica Femina Centra—Polyclinic of Gynecology and Senology, Outpatient Gynecologic and Senologic Clinic “Wolf”, Srebrna 46/5, 80-180 Gdansk, Poland; 4 Transplant and General Surgery, Department of Surgical Oncology, Medical University of Gdansk, Smoluchowskiego 17, 80-210 Gdansk, Poland; omusielak@gumed.edu.pl; 5 Department of Biomedical Sciences, College of Health Sciences, Abu Dhabi University, Abu Dhabi P.O. Box 59911, United Arab Emirates; 6 Faculty of Medicine, University of Ahmed Ben Bella, Oran 31000, Algeria; yousrasafari@ymail.com; 7 Division of Medical Oncology, Department of Oncology, Schulich School of Medicine & Dentistry, Western University, London, ON N6A 5C1, Canada; 8 Verspeeten Family Cancer Centre, London Health Sciences Centre, London, ON N6A 5W9, Canada

**Keywords:** HPV, cervical cancer, vaccination barriers, vaccination coverage

## Abstract

**Background/Objectives**: Cervical cancer is the second most common gynecological cancer worldwide, preventable through screening initiatives and vaccinations against its causative agent, anogenital human papillomavirus (HPV). This study aimed at measuring the coverage and uptake of the national HPV vaccination program launched in 2023 and implemented throughout Poland. **Methods**: This cross-sectional, observational study analyzed population data of adolescents in 11–13-year-old groups vaccinated in individual voivodeships (provinces) of Poland as provided by the National Health Fund and the Central Statistical Office. A *p*-value of <0.05 was considered statistically significant. **Results:** The rate of HPV vaccination participation under the population program was 8.67%. In the analyzed age groups, in both sexes, no statistically significant correlation was observed between the population size at a given age and population coverage or participation in HPV vaccination. However, a positive relationship in vaccination coverage was observed in individuals previously vaccinated with one dose in subsequent age groups, indicating a continued willingness to receive vaccination with further doses. No statistically significant difference in population coverage changes across voivodeships was found between the number of doses within the urban population share vs. rural population share. **Conclusions**: Our results show that, at 1.5 years of implementation of the national HPV vaccination program, the coverage and uptake of the program is considerably insufficient. The intensive corrective actions indicated are required to pave this program forward towards optimum results.

## 1. Introduction

Vaccinations serve as a source of long-term protection and prevention against various diseases. The significance of vaccination in the robust eradication of infectious agents is considered a paradigm shift, saving millions of lives worldwide [1,2].

Cervical cancer is the fourth most common gynecological cancer in women worldwide, with approximately 600,000 new cases and 350,000 deaths reported globally in year 2022 [3,4]. The major causative agent for cervical cancer (approximately 80% of the cases) is attributed to human papillomavirus (HPV). Moreover, this virus has also been associated with benign genital warts, and vulvar, vaginal, anal, penile, and laryngeal cancers. Fortunately, robust screening and vaccination initiatives against both low-risk (LR) and high-risk (HR) HPV virus strains have proved to serve as robust preventive measures, helping to reduce the burden of cervical as well as its associated cancers globally [5,6,7]. Among the high-risk (HR) HPV types, HPV-16 and -18 are considered highly carcinogenic due to their expression of oncoproteins E6 and E7. These viral oncoproteins initiate uncontrolled cell cycle progression via the disruption of signaling pathways associated with p53 and pRB tumor suppressor proteins. Persistent HR HPV infection with overexpression of E6 and E7 initially leads to precancerous changes in the cervical epithelium, known as cervical intraepithelial neoplasia (CIN), that further progress to invasive cervical cancer, ultimately spreading to the uterus, bladder, rectum, and pelvic lymph nodes [8].

Regardless of an individual’s HPV test result (positive or negative), or history of cervical conization, vaccination against HPV has been reported to increase the local immune response against the virus, thus preventing disease recurrence by eradicating viral replication [9]. Worldwide, HPV eradication through vaccinations has clearly demonstrated an advantage via cancer risk reduction, consequently leading to a reduction in treatment and hospital-associated burden on both the government and the affected individuals [10,11].

According to the Catalan Institute of Oncology/International Agency for Research on Cancer (ICO/IARC) data, Poland has reported that approximately 3.4% of cervical infection cases in women are associated with HPV-16 and/or HPV-18. On the other hand, 88.1% of reported invasive cervical cancers have been attributed to HPV-16 or -18 strains, indicating the high risk associated with the respective HPV types [12].

In 2020, the WHO adopted the 90–70–90 target for 2030 that aims to reduce the incidence of HPV-associated cervical cancers. The target is geared towards the following goals: 90% of girls to be fully vaccinated with the HPV vaccine by the age of 15, 70% of women screened using a high-performance test by the age of 35, and again by the age of 45, and lastly, 90% of women with pre-cancerous lesions treated and 90% of women with invasive cancer managed with minimally invasive interventions [13]. According to this WHO strategy, a two-dose HPV vaccination has been recommended if the first dose is administered under 15 years of age. Several countries have already adopted this strategy in the context of compliance and cost reduction [14,15]. In recent years, however, sentiment around population-based HPV vaccination has cooled somewhat. In Japan, HPV vaccination has been suspended in recent years due to adverse events (such as cerebral vasculitis), which in turn has caused a decline in HPV vaccination coverage in other countries participating in the WHO program [16]. It is worth noting, that WHO, along with non-WHO institutions (like The Bill & Melinda Gates Foundation), fight for enhancing attitudes around HPV vaccines. There have also been studies suggesting that vaccination with a single dose of the vaccine may be sufficient [17].

Furthermore, clinical trials, large-scale studies, and real-world data have documented that the effectiveness of the HPV vaccine, with one or more doses administered, ranges from 83.0% to 96.1%. On the other hand, an approximate 90% reduction in type 6, 11, 16, and 18 HPV infections and a nearly 90% reduction in cervical cancer incidence among girls vaccinated before the age of 17 have been reported in several countries that adopted the WHO recommendations on vaccination strategy [18,19]. Similarly, the quadrivalent HPV vaccine has been reported to effectively prevent high-grade cervical lesions, supporting its role in preventing invasive cervical cancer [19,20,21].

In Poland, population-based HPV vaccination was launched as a national immunization program in June 2023 with the Cervarix^®^ (quadrivalent anti-HPV vaccine, GlaxoSmithKline Biologicals S.A., Rixensart, Belgium) and Gardasil 9^®^ (nine-valent anti-HPV vaccine, Merck Sharp & Dohme B.V., Haarlem, The Netherlands) vaccines available free of cost to the public [22,23]. At the end of August 2023, a total of 83,782 teenagers were vaccinated (65% girls, 35% boys, with an age bracket of 12–13 years) within the free HPV vaccination program. The vaccination share was at 9.8%, which was considered low [24]. According to Eurostat, administrative data from cervical screening programs available for 20 European Union (EU) countries, cervical cancer screening percentage has ranged from 78.8% in Sweden to 4.5% in Romania. Poland ranked in the second position from the very end in this statistic, only surpassing Romania with a 10.9% screening rate [25].

Keeping in perspective the low coverage rates in Poland, observed in August 2023, this study aimed to measure the range and effectiveness of the national HPV vaccination program nearly 1.5 year after its implementation in Poland. Moreover, the study analyzes correlations between the HPV vaccine doses administered and factors, including population size, population shares (urban/rural) coverages, regional variations, and temporal changes associated with the immunization program. The study will provide insight into the low immunization rates, and suggest solutions for both Poland as well as for other countries that have not implemented nationwide vaccination programs.

## 2. Materials and Methods

### 2.1. Study Design

This observational, cross-sectional study analyzed population-based data on the national HPV vaccination program, collected through the National Health Fund (NFZ) and Central Statistical Office (GUS). The data comprised HPV vaccinations (Cervarix^®^ or Gardasil-9^®^) administered from 2023 to October 2024 in 16 voivodeships (provinces) and the entire country to adolescents in three age groups (11, 12, and 13 years old). The English names of Polish local government units, called voivodeships, are provided in the manuscript, in accordance with the guidelines of the Commission for the Standardization of Geographical Names Outside the Republic of Poland.

Both the NFZ and GUS granted the authors’ request to share the data in accordance with the principles of access to public information. The data obtained was compared with the public state website—report on vaccinations against human papillomavirus (HPV) available at https://ezdrowie.gov.pl/portal/home/badania-i-dane/raport-o-szczepieniach-przeciwko-wirusowi-brodawczaka-ludzkiego-hpv (accessed on 4 August 2025).

HPV vaccination programs in Poland administer a two-dose regimen as part of the vaccination initiative. For the study, a vaccinated individual was considered a patient in a given age group who received at least one dose of HPV vaccination exclusively as part of the program and reported it in their vaccination record (based on the International Classification of Medical Procedures (ICD-9)—code 99.559).

### 2.2. Statistical Analysis

The statistical significance for all the tests was set at α = 0.05, corresponding to a 5% probability of type I error (false positivity). No data transformations were applied, as variables were continuous and within plausible ranges. Normality was assessed using a Kolmogorov–Smirnov test. Since several variables did not meet the assumption of normality, nonparametric methods were applied: Spearman’s rank correlation to assess associations between variables, while group differences were examined using the Kruskal–Wallis test.

## 3. Results

The results obtained from the analysis were divided into five sections, presented below.

### 3.1. Relationship Between the Number of Vaccinations and Population Share, and Relationship Between the Number of Vaccinations and Urban Population Shares

**Figure 1:**
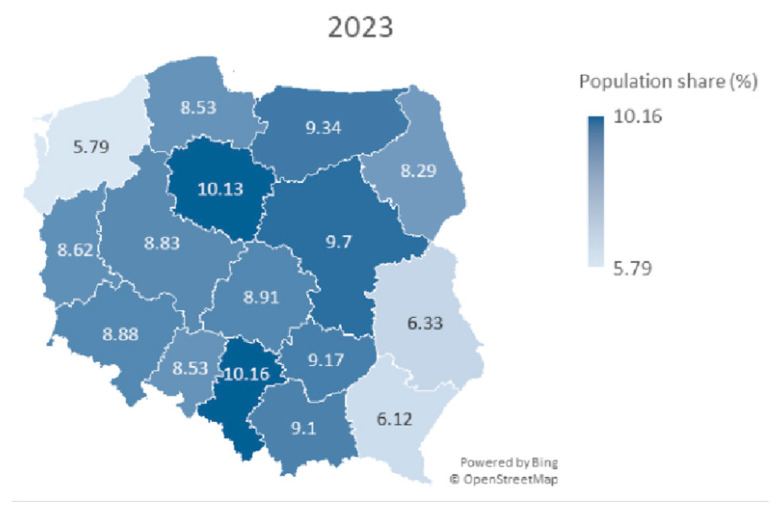
A map of Poland showing population share (%) of HPV vaccines among children aged 11–13 years for both sexes in particular voivodeships in year 2023.

At the end of the observation period (October 2024), the level of participation in HPV vaccination, under the population program, was observed to be 8.67%, which is slightly lower than the overall coverage of HPV vaccination in Poland reported by state authorities (9.09%). This result indicates that, regardless of age or the source of financing for the vaccination, inclination for receiving HPV vaccinations remains similar in the Polish society. Poland is divided into sixteen administrative units called voivodships. The level of participation in HPV in voivodships varied from 5.48% to 11.48% (Figure 1).

Sperman’s correlation coefficient was used to check the level of association between the number of vaccinations and the percent of target population share in voivodeships. In 2023, the coefficient was *r_s_* = 0.606 (*p* = 0.013), and in 2024, *r_s_* = 0.406 (*p* = 0.119), indicating a statistically significant, positive correlation in 2023, but not in 2024. In 2023, the greater number of vaccinations was correlated with greater population share in the regions. In 2024, this effect decreased. Furthermore, it implies that higher doses do not necessarily correspond to higher vaccination coverage rates.

**Figure 2:**
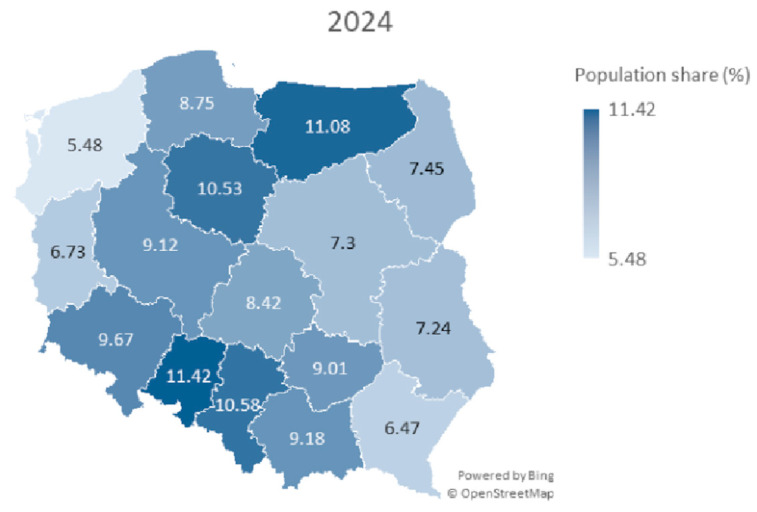
A map of Poland showing population share (%) of HPV vaccines among children aged 11–13 years for both sexes in particular voivodeships in year 2024.

Further analysis considered the correlation between the number of vaccinations in 2023 and 2024 and urban shares for the regions. Spearman’s coefficient was *r_s_* = 0.06, *p* = 0.811, for urban population share. This does not allow us to reject the hypothesis about the lack of correlation between the number of vaccinations and the proportion of the urban population (Table 1). The map of Poland with marked voivodeships and % population coverage with HPV vaccination in selected periods of 2023 and 2024 is shown in Figure 1 and Figure 2. The map shows a pattern in which the eastern part of Poland has lower HPV vaccination coverage.

### 3.2. Correlation Between Population Size and Population Percent Coverage in HPV-Vaccinated Individuals Across 16 Voivodeships in Poland for Three Distinct Age Groups (11–13 Years Old)

The data presented in Table 2 was used to calculate Spearman’s coefficients (and the corresponding t-test for these coefficients), as well as the Kruskal–Wallis test for the variable coverage change. The purpose of applying the Kruskal–Wallis test was to verify the hypothesis that the level of changes did not differ statistically across voivodeships.

In both 2023 and 2024, the correlation between the variable of interest and the 11-year-old age group was negative but not statistically significant (2023: r = −0.177, *p* = 0.513; 2024: r = −0.009, *p* = 0.974). Therefore, there is insufficient evidence to conclude the existence of a meaningful correlation. In both 2023 and 2024, the correlation between the variable of interest and the 12-year-old age group was weak and statistically non-significant (2023: r = 0.025, *p* = 0.927; 2024: r = 0.294, *p* = 0.268). Thus, there is no sufficient evidence to support the existence of a meaningful correlation. For the 13-year-old age group, correlations were r =0.233 (*p* = 0.387) in 2023 and r = 0.256 (*p* = 0.339) in 2024, indicating negligible, non-significant correlation, indicating no clear relationship between population size and vaccination coverage.

Comparison between both sexes in the age groups of 11, 12, and 13 years old showed no statistically significant correlation between the size of the population at a given age and population coverage/participation in HPV vaccination initiative.

### 3.3. Relationship Between Changes in Percentage Population and Changes in Population Percentage from 2023 to 2024 for the Same Age Groups

The relationship between population percent changes and population shares percent changes from 2023 to 2024 was assessed using Spearman’s correlation coefficients across the 16 voivodships, stratified by age (Table 2). For the 11-year-old age group, the correlation coefficient was r = 0.027 (*p* = 0.922), indicating a lack of statistically significant correlation. For the 12-year-old age group, the correlation coefficient was r = −0.358 (*p* = 0.173), showing no statistically significant correlation. For the 13-year-old age group, the correlation coefficient was r = −0.097 (*p* = 0.720), reflecting lack of significant correlation.

The flow of people previously vaccinated with one dose to subsequent age groups indicate a non-significant correlation in vaccination coverage, which may mean that a person vaccinated in the previous age group with one dose does not increase interest in starting vaccination of peers in subsequent age groups.

### 3.4. Analysis of Regional Variations in HPV Vaccination Coverage Changes for the 13-Year-Old Age Group in Poland, 2023–2024

This sub-analysis aimed to determine whether the increase in HPV vaccine doses administered and the population coverage percent change for the 13-year-old age group are equal in proportion across 16 voivodeships in Poland from 2023 to 2024. The average percentage change for Poland in this age group was 37.9% (from 9.7% coverage in 2023 to 13.4% coverage in 2024).

**Figure 3:**
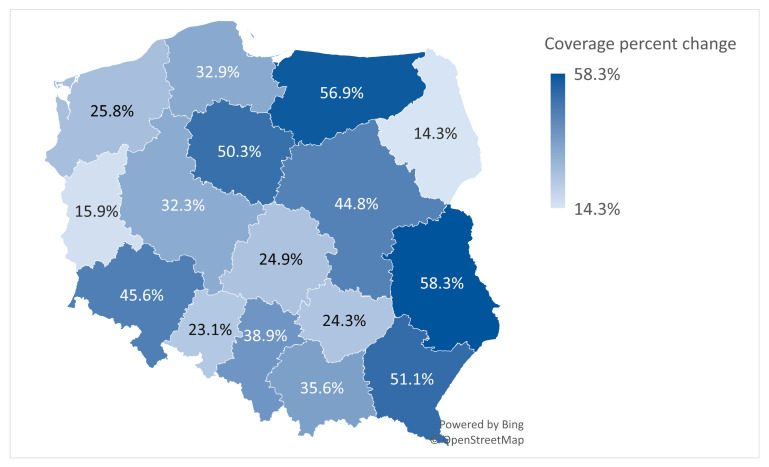
The population coverage percent change for the 13-year-old age group.

The Lubelskie voivodeship had the greatest increase in population HPV vaccination coverage percent change, with 58.3%, reflecting the largest relative increase in vaccination coverage. The voivodeship with the most diminished (lowest) change was Podlaskie (14.3%), indicating the smallest relative increase in HPV vaccination coverage. Other notable changes include the Warminsko-Mazurskie voivodeship, located in the North-East of Poland (56.9%), which was the second-highest, and Podkarpackie, situated in the South-East (51.1%), the third-highest, while Lubuskie (15.9%) and Opolskie (23.1%) were among the lowest (Figure 3).

Analysis with a Kruskal–Wallis test yielded a test statistic of H = 15.0 (df =15, *p* = 0.45), providing evidence that the population percentage changes do not differ across voivodships (*p* > 0.05). Given the small sample size (n = 16) and single observation per voivodship, the test’s power is limited and is statistically insignificant.

### 3.5. Comparison of HPV Vaccination Doses and Coverage by Sex and Age Groups in Poland, 2023–2024

Overall, the findings reveal modest vaccination coverage rates (a maximum of 16.6% in the group of 13-year-old females in 2024), as well as temporal variations between the 2023 and 2024. Females consistently exhibited higher coverage compared to males across all strata. Age-related patterns suggest that coverage tends to increase with advancing age within each year.

Sex gaps persisted (female-to-male ratios 1.35–2.1-fold), and were widest at age 12 in 2023 (15.74% vs. 8.18%) and age 13 in 2024 (16.60% vs. 10.32%), which is attributable to female-centric perceptions of HPV risks despite guidelines for gender-neutral vaccination to mitigate diverse cancers (Table 3).

## 4. Discussion

This study presents an analysis of the participation of children aged 11–13 in the free-of-charge, nationwide HPV vaccination program in Poland over 1.5 years following its introduction, using data from the NHF-CSO (since June 2023). This is the first assessment of the performance of the vaccination program in Poland. The estimated global vaccination rate coverage of the HPV vaccine remains low (12%). In the World Health Organization (WHO) European Region, the prevalence is approximately 31%. For the “EU-15” countries (this refers to the 15 European Union member states that existed before the major enlargements in 2004), it is correspondingly higher, e.g., Belgium—90%, Great Britain—85%, Denmark—80%, Sweden—80% [26,27]. However, there are developing countries in the world, such as most Arab countries, where public awareness of HPV and vaccination is insufficient and there are no widespread HPV vaccination programs [28,29].

The study demonstrated that there is no evidence that larger populations are associated with higher population coverage with the HPV vaccine. Voivodeships with low population growth or decline tend to experience fewer negative changes in population structure. This suggests that demographic stability can significantly limit the decline in HPV vaccination rates. Voivodeships experiencing population growth or slight decline are more likely to experience milder demographic changes, which may help maintain higher vaccination rates or slow down their decline.

Innovative strategies, such as integrating adolescent HPV vaccination with cervical cancer screening in women aged 30–45, may be beneficial to increase the/vaccination rate and accelerate HPV elimination in Poland. Improving vaccination rates among children and adolescents requires not only easy systemic vaccine access, but it also needs to address parents’ concerns about the safety and effectiveness of the HPV vaccines [26]. An unwillingness to recommend HPV vaccines [30] as well as a dangerous vaccine hesitancy trend have been observed among medical professionals [31,32]. More intensive communication strategies, including school-based education, are necessary to improve parents’ state of knowledge about diseases associated with HPV and promote vaccination decision-making in adolescents. Parental education is another proven factor influencing willingness to vaccinate a child against HPV. The higher the parents’ education level, the larger the vaccination supporters’ group. Improved declared parental knowledge about the risk of HPV infection was found to be associated with greater trust in the physicians and higher willingness to vaccinate the child against HPV [33,34].

In the studied group of adolescents, a lower percentage of vaccinations was represented by the male gender. Unfortunately, the social norms perpetuated over several years, vaccine myths [35], and national policy contributed to the fact that the HPV vaccine is still considered a “feminine” vaccine, associated mainly with girls and women [36]. Daley et al. pointed out that the feminization of the HPV vaccine may negatively impact HPV-related health prophylaxis [37]. Men are also at risk of HPV-related neoplasms; however, this threat is poorly understood among Polish men. A comprehensive educational effort should be implemented to raise awareness among boys and their parents (particularly fathers), to reinforce the belief that HPV vaccination affects them in equal measure to the vaccination of girls and women [38]. Another group that should be the target of educational programs about the risks and routes of HPV infection are the LGBTQ+ community. Adopting a gender-neutral approach to HPV vaccination will reduce the number of HPV infections and diseases transmitted among the population, combat disinformation and fake news, and minimize vaccine-related stigma and promote gender equality [39].

Some studies demonstrate that rural areas are significantly more susceptible to exclusion from healthcare, including vaccinations. This is due to limited access to healthcare services, higher costs associated with traveling to the nearest medical facility, and awareness issues [40]. Despite these obvious arguments, HPV vaccination rates in rural areas may not be significantly lower. In our study, we did not find a significantly higher share of HPV vaccinations in voivodeships with a larger share of urban areas. A survey by Sikora et al. of teens from urban and rural areas in the United States found that rural girls had lower rates of complete HPV vaccination than their urban counterparts [41]. However, reports from 2023 from Vietnam suggest that the overall vaccination rate was 4%, with urban women having a higher rate of 4.9% compared to rural women at 3.1% [42].

Standardized rates of cervical cancer morbidity in Poland (Incidence rates—ESP2013) indicate that, in 2021, the highest incidence was observed in the following voivodeships: Swietokrzyskie (13.3), Warminsko-Mazurskie (12.8), and Pomorskie (12.5). The lowest incidence was observed in the following voivodeships: Podkarpackie (7.7), Malopolskie (8.2), and Lubelskie (8.3). The average for Poland was 10.4 [43].

Interestingly, the Pomorskie Voivodeship has one of the highest HPV vaccination rates for the entire population (within the population program, and outside the program—private funding, for all age groups and genders) at 19.12%, with a national average of 15.92%. Meanwhile, the Podkarpackie Voivodeship, with one of the lowest cervical cancer incidence rates, had the lowest population vaccination rate at 9.8%, using the same criteria.

Further research could focus on patterns of sexual behavior or the incidence of other HPV-related cancers in these voivodeships.

We demonstrated a lower level of coverage with HPV vaccination during the observation period in the eastern regions of Poland, which fits into the broader context of the propensity to vaccinate in Poland. In eastern Poland, the average percentage of children up to 3 years of age who are not vaccinated at all according to the mandatory vaccination schedule is significantly higher, and amounts to 2.12%, with the average in Poland being 1.5% [44].

Despite the efforts and financial outlays on HPV vaccination, the first results of the implementation should be considered far from satisfactory. It is striking that subsequent countries, including Poland, launching vaccination programs do not focus on proven criteria responsible for the success of implementation: awareness and positive attitude of parents towards the HPV vaccination program, mobilization of healthcare professionals, and coordinated strategy [33,45,46].

To improve the effectiveness of national HPV vaccination programs, it seems appropriate to highlight the following recommendations:

(a) Patient interest in publicly funded HPV vaccinations is low in countries without long-standing vaccination awareness. Urgent, widespread outreach efforts should be initiated using social media and involving influencers.
(b) It is expected that vaccination success will not be achieved without the involvement of medical professionals. Training programs for doctors and nurses are needed to develop them as ambassadors for HPV vaccination. Given the shortage of medical staff, an additional financial bonus program “for success” seems essential.
(c) Decision-makers should develop a program to achieve the assumed goal of population coverage with HPV vaccination at three levels: strategic—responsible for creating the mission and vision for the entire process; management—accountable for human resources, IT support, communication model, and corrective actions; and operational—day-to-day implementation of the process.

## 5. Conclusions

These preliminary results indicate that, after the first 1.5 years of implementing the national HPV vaccination program, its impact is insufficient. Although this is a step in the right direction, the results so far are not promising. The program requires intensive corrective actions. Our research may be valuable for decision-makers in conducting more informative, widely accessible campaigns promoting HPV vaccines—in traditional and social media, where young people are particularly active. Another group that requires a refresher on HPV is physicians. They play a key role in encouraging vaccination, as they encounter patients and their parents during medical visits. The analysis of the initial implementation results we presented may serve as a guide for other countries planning to launch HPV vaccination programs and prevent the same early mistakes from being made in the future. The direction we have presented seems promising, especially in the context of creating a model for an HPV vaccination program that will be resistant to the limitations identified in our study.

## 6. Study Limitations

The main limitation of this study is the short period spent on the observation and monitoring of vaccinations since the official free-of-charge HPV vaccination program was applied in Poland. The study only considers administered doses of HPV vaccines, not the number of individuals who are completely vaccinated, which, in turn, drives to generalizations and insufficient precision in drawing conclusions based on publicly available data. We were interested in a targeted study at the national level to provide a detailed and independent assessment of immunization uptake based on the available data; however, the study lacks a report on the interest in HPV immunization, as well as data on the implementation of the HPV vaccination program at the national level. We also did not analyze a larger population than just 11–13-year-olds. However, the 11–13-year-old population may be more aware than younger groups and more decisive, being the target population for immunization programs. Further research will focus on the direct causes of low vaccine uptake among the 11–13-year-old population, given the current widespread access to vaccines in Poland.

## Figures and Tables

**Table 1:** HPV vaccinations as part of a population program among children aged 11–13 years for both sexes by voivodeship in Poland, June–December 2023 to January–October 2024: population and urban/rural distribution.

Voivodeship	Year	Number of Vaccinations	Target Population (N)	Target Population Share (%)	Urban Target Population Share (%)	Rural Target Population Share (%)
Wielkopolskie	2023	10,634	120,488	8.83	46.85	53.15
	2024	10,800	118,392	9.12	46.69	53.31
Swietokrzyskie	2023	3330	36,320	9.17	41.28	58.72
	2024	3193	35,435	9.01	41.20	58.80
Kujawsko- Pomorskie	2023	6681	65,944	10.13	51.55	48.45
	2024	6806	64,645	10.53	51.61	48.39
Malopolskie	2023	10,508	115,483	9.10	40.67	59.33
	2024	10,465	113,956	9.18	40.63	59.37
Dolnoslaskie	2023	7877	88,712	8.88	60.86	39.14
	2024	8414	87,002	9.67	60.59	39.41
Lubelskie	2023	4170	65,839	6.33	42.03	57.97
	2024	4662	64,390	7.24	42.08	57.92
Lubuskie	2023	2795	32,417	8.62	60.86	39.14
	2024	2141	31,817	6.73	60.77	39.23
Mazowieckie	2023	15,997	164,861	9.70	60.34	39.66
	2024	13,495	184,901	7.30	60.56	39.44
Opolskie	2023	2378	27,870	8.53	50.05	49.95
	2024	3150	27,591	11.42	49.69	50.31
Podlaskie	2023	3017	36,378	8.29	57.75	42.25
	2024	2661	35,705	7.45	57.53	42.47
Pomorskie	2023	6926	81,182	8.53	54.24	45.76
	2024	6986	79,847	8.75	54.10	45.90
Slaskie	2023	13,982	137,671	10.16	72.49	27.51
	2024	14,315	135,355	10.58	72.33	27.67
Podkarpackie	2023	4233	69,183	6.12	37.37	62.63
	2024	4418	68,280	6.47	37.31	62.69
Warminsko- Mazurskie	2023	4235	45,342	9.34	54.82	45.18
	2024	4914	44,368	11.08	54.75	45.25
Zachodnio- pomorskie	2023	2936	50,684	5.79	63.47	36.53
	2024	2729	49,792	5.48	63.31	36.69
Lodzkie	2023	6577	73,833	8.91	56.43	43.57
	2024	6093	72,348	8.42	56.51	43.49
Poland (total)	2023	106,276	1,212,207	8.77	53.19	46.81
	2024	105,242	1,213,824	8.67	53.10	46.90

Annotations: Number of Vaccinations represents the number of human papillomavirus (HPV) vaccinations administered as part of the population vaccination program for the specified year and age group, target population sizes in age group 11–13 years (N), and percentages (Target population Share, Urban Target Population Share, Rural Target Population Share). Target Population Share (%) is calculated as (Number of Vaccinations/Population) × 100. Urban and Rural Target Population Shares (%) represent the proportion of the population in urban and rural areas, respectively, summing to 100%. All data is complete with no missing values. The table is designed to accommodate future stratification by sex and age groups, with current data aggregated across these demographics for 2023 and 2024.

**Table 2:** Changes in HPV vaccination administration and population dynamics by voivodship and age group in Poland, years 2023–2024 (examined period).

Voivodship	Age Group	Number of Vaccinations		Target Population			Target Population Coverage (%)		Coverage Change
		2023	2024	2023	2024	Change (%)	2023	2024	%2024–%2023
Wielkopolskie	11-year	1211	923	39,427	38,535	−2.3	3.07	2.40	−0.67
	12-year	4836	3998	39,225	39,317	0.2	12.33	10.17	−2.16
	13-year	4587	5879	41,836	40,540	−3.1	10.96	14.50	−3.54
Swietokrzyskie	11-year	395	287	11,764	11,340	−3.6	3.36	2.53	−0.83
	12-year	1496	1177	11,864	11,822	−0.4	12.61	9.96	−2.65
	13-year	1439	1729	12,692	12,273	−3.3	11.34	14.09	−2.75
Kujawsko-Pomorskie	11-year	902	567	21,461	20,962	−2.3	4.20	2.70	−1.5
	12-year	3227	2539	21,447	21,465	0.1	15.05	11.83	−3.22
	13-year	2552	3700	23,036	22,218	−3.6	11.08	16.65	−5.57
Malopolskie	11-year	1371	873	37,860	37,238	−1.6	3.62	2.34	−1.28
	12-year	4917	3992	37,955	37,898	−0.2	12.95	10.53	−2.42
	13-year	4220	5600	39,668	38,820	−2.1	10.64	14.43	−3.79
Dolnoslaskie	11-year	937	692	28,856	28,121	−2.5	3.25	2.46	−0.79
	12-year	3663	3089	29,035	28,953	−0.3	12.62	10.67	−1.95
	13-year	3277	4633	30,821	29,928	−2.9	10.63	15.48	−4.85
Lubelskie	11-year	650	556	21,430	20,726	−3.3	3.03	2.68	−0.35
	12-year	1972	1727	21,541	21,478	−0.3	9.15	8.04	−1.11
	13-year	1548	2379	22,868	22,186	−3.0	6.77	10.72	3.95
Lubuskie	11-year	375	142	10,630	10,310	−3.0	3.53	1.38	−2.15
	12-year	1358	803	10,575	10,614	0.4	12.84	7.57	−5.27
	13-year	1062	1196	11,212	10,893	−2.8	9.47	10.98	1.51
Mazowieckie	11-year	1278	1331	61,622	60,447	−1.9	2.07	2.20	0.13
	12-year	5599	5417	61,127	61,403	0.5	9.16	8.82	−0.34
	13-year	4798	6747	64,914	63,051	−2.9	7.39	10.70	3.31
Opolskie	11-year	312	235	9285	8991	−3.2	3.36	2.61	−0.75
	12-year	1122	889	9100	9195	1.0	12.33	9.67	−2.66
	13-year	944	1139	9485	9296	−2.0	9.95	12.25	2.3
Podlaskie	11-year	387	262	11,950	11,580	−3.1	3.24	2.26	−0.98
	12-year	1348	973	11,883	11,911	0.2	11.34	8.17	−3.17
	13-year	1282	1426	12,545	12,214	−2.6	10.22	11.68	1.46
Pomorskie	11-year	802	638	26,579	25,992	−2.2	3.02	2.45	−0.57
	12-year	3253	2643	26,460	26,538	0.3	12.29	9.96	−2.33
	13-year	2871	3705	28,143	27,317	−2.9	10.20	13.56	3.36
Slaskie	11-year	1615	1196	44,997	44,009	−2.2	3.59	2.72	−0.87
	12-year	6555	5263	45,021	45,011	0.0	14.56	11.69	−2.87
	13-year	5812	7856	47,653	46,335	−2.8	12.20	16.95	4.75
Podkarpackie	11-year	612	365	22,739	22,275	−2.0	2.69	1.64	−1.05
	12-year	1943	1561	22,811	22,770	−0.2	8.52	6.86	−1.66
	13-year	1678	2492	23,633	23,235	−1.7	7.10	10.73	3.63
Warminsko-Mazurskie	11-year	539	560	14,667	14,228	−3.0	3.67	3.94	0.27
	12-year	2018	1783	14,977	14,812	−1.1	13.47	12.04	−1.43
	13-year	1678	2571	15,698	15,328	−2.4	10.69	16.77	6.08
Zachodniopomorskie	11-year	470	335	16,572	16,251	−1.9	2.84	2.06	−0.78
	12-year	1434	1146	16,392	16,484	0.6	8.75	6.95	−1.80
	13-year	1032	1248	17,720	17,057	−3.7	5.82	7.32	1.50
Lodzkie	11-year	929	458	24,159	23,341	−3.4	3.85	1.96	−1.89
	12-year	3094	2363	24,202	24,181	−0.1	12.78	9.77	−3.01
	13-year	2554	3272	24,202	24,826	2.6	10.55	13.18	2.63

Notes: Number of Vaccination represents the number of human papillomavirus (HPV) vaccinations administered as part of the population vaccination program for the specified year and age group. Target population sizes in age group 11–13 years (N), Target Population Coverage (%) represtents ratio of the number of vaccinations to the target population ×100.

**Table 3:** Summary of product settlement patterns among children aged 11–13 years, stratified by year, age, and sex, for 2023 and 2024.

Year	Age (Years)	Sex	Number of Vaccination	Population (N)	Population Share (%)
2023	11	Female	8502	196,262	4.33
2023	11	Male	4283	207,736	2.06
2023	12	Female	30,852	196,054	15.74
2023	12	Male	16,983	207,561	8.18
2023	13	Female	24,942	207,713	12.01
2023	13	Male	16,392	219,683	7.46
2024	11	Female	5912	191,764	3.08
2024	11	Male	3508	202,582	1.73
2024	12	Female	25,396	196,160	12.95
2024	12	Male	13,967	207,692	6.72
2024	13	Female	33,521	201,915	16.60
2024	13	Male	22,051	213,602	10.32

Annotations: Data is presented as counts (Number of Vaccinations), population sizes (N), and percentages (Population Share). Population Share (%) is calculated as (Number of Vaccinations/Population) × 100. All data is complete with no missing values. The table summarizes product settlement patterns among children aged 11–13 years, stratified by year, age, and sex, for 2023 and 2024.

## Data Availability

Data is available as public information at the National Health Fund and the Central Statistical Office, and additionally at https://ezdrowie.gov.pl/portal/home/badania-i-dane/raport-o-szczepieniach-przeciwko-wirusowi-brodawczaka-ludzkiego-hpv (accessed on 4 August 2025).

## Abbreviations

HPV	Human papillomavirus
WHO	World Health Organization
HR HPV	High-risk human papillomavirus
LR HPV	Low-risk human papillomavirus
E6, E7	Oncoproteins of HPV
p53	Cellular tumor antigen 53; tumor suppressor protein
pRB	Retinoblastoma protein; tumor suppressor protein
CIN	Cervical intraepithelial neoplasia
IARC	International Agency for Research on Cancer
EU	European Union
NHF	National Health Fund
CSO	Central Statistical Office
LGBTQ+	Lesbian, Gay, Bisexual, Transgender, and Queer/Questioning, other sexual orientations
